# Clinical and Biochemical Features Associated with Coexisting Primary Aldosteronism in Patients with Obstructive Sleep Apnea: A Cross-Sectional Study

**DOI:** 10.3390/diagnostics16162565

**Published:** 2026-08-14

**Authors:** Mingliu Li, Xuyong Chen, Yi Zhang, Yuanyuan Teng, Biling Huang, Min Tan, Zehao Liu, Min Guo, Chun Li, Xiaoli Su, Tiejian Jiang, Min Wang

**Affiliations:** 1 Department of Endocrinology, Xiangya Hospital, Central South University, Changsha 410008, China; yljiajia@126.com (M.L.); chenxuyong2022@163.com (X.C.); 248112189@csu.edu.cn (Y.T.); 258102097@csu.edu.cn (B.H.); tanmin961119@sina.com (M.T.); zehaoliu@csu.edu.cn (Z.L.); xyguom@hotmail.com (M.G.); xylichun27@163.com (C.L.); jiangtj1971@163.com (T.J.); 2 National Clinical Research Center for Geriatric Disorders, Xiangya Hospital, Central South University, Changsha 410008, China; zhangyi0205@csu.edu.cn (Y.Z.); suli8779@163.com (X.S.); 3 Department of Geriatric Endocrinology and Metabolism, Guangxi Academy of Medical Sciences, The People’s Hospital of Guangxi Zhuang Autonomous Region, Nanning 530021, China; 4 Department of Orthopaedics, Xiangya Hospital, Central South University, Changsha 410008, China; 5 Department of Respiratory, Xiangya Hospital, Central South University, Changsha 410008, China; 6 Department of Endocrinology, Peking University Shenzhen Hospital, Shenzhen 518036, China

**Keywords:** obstructive sleep apnea, primary aldosteronism, aldosterone, serum potassium, cross-sectional study

## Abstract

**Background:** Obstructive sleep apnea (OSA) and primary aldosteronism (PA) are linked by bidirectional mechanisms. Although guidelines recommend PA screening in hypertensive patients with OSA, simple tools for clinical prioritization remain limited. We aimed to characterize clinical, polysomnographic, and biochemical features associated with coexisting PA in patients with OSA. **Methods:** We conducted a cross-sectional study in 107 adults with OSA and definitive PA classification, including 30 patients in the OSA+PA group and 77 in the OSA-only group. Clinical, polysomnographic, and fasting biochemical variables were collected using standardized procedures. Multivariable logistic regression was used to examine factors associated with coexisting PA. **Results:** Compared with the OSA-only group, the OSA+PA group had a larger neck circumference (42.0 ± 4.6 vs. 39.8 ± 3.7 cm, *p* = 0.010), a higher snoring index (369.9 ± 320.3 vs. 252.0 ± 264.9, *p* = 0.040), and higher aldosterone levels (25.8 ± 18.8 vs. 10.7 ± 7.3 ng/dL, *p* = 0.001), while glycated hemoglobin was lower (6.4 ± 1.6 vs. 7.5 ± 2.1, *p* = 0.005) and serum potassium was significantly reduced (3.5 ± 0.4 vs. 4.0 ± 0.4 mmol/L, *p* = 0.001). In multivariable analysis, aldosterone (OR = 1.138, *p* = 0.001) and serum potassium (OR = 0.017, *p* = 0.001) were associated with coexisting PA. **Conclusions:** Higher aldosterone and lower serum potassium were readily available biochemical features associated with coexisting PA among clinically selected patients with OSA.

## 1. Introduction

Obstructive sleep apnea (OSA) is a common sleep-related breathing disorder characterized by recurrent upper-airway collapse, intermittent hypoxemia, and sleep fragmentation [1]. Its prevalence is increasing worldwide, particularly among individuals with obesity and hypertension, and it is associated with increased cardiovascular morbidity and mortality [2]. Primary aldosteronism (PA), a common cause of secondary hypertension, is characterized by autonomous aldosterone production, sodium retention, renin suppression, and, in some patients, hypokalemia. Compared with essential hypertension, PA confers additional cardiovascular and renal risk beyond that attributable to blood pressure alone [3].

Evidence suggests a bidirectional relationship between OSA and PA [4]. Intermittent hypoxia and sustained sympathetic activation in OSA may stimulate the renin–angiotensin–aldosterone system and contribute to nocturnal and early-morning blood pressure elevation, often manifesting as non-dipping or resistant hypertension [2,5]. Conversely, aldosterone-mediated sodium and fluid retention may promote rostral fluid redistribution, pharyngeal edema, and worsening upper-airway obstruction [2,6]. Accordingly, current guidelines recommend PA screening in hypertensive patients with OSA [7,8]. However, implementation in routine practice remains limited, and coexisting PA may remain underrecognized.

In this context, we conducted a cross-sectional study to characterize clinical, polysomnographic, and biochemical features associated with coexisting PA among patients with OSA.

## 2. Materials and Methods

### 2.1. Research Design and Participants

We conducted a retrospective cross-sectional study in the Department of Endocrinology of Xiangya Hospital from 2021 to 2022. Consecutive patients aged 18–75 years who underwent overnight polysomnography because of clinical suspicion of obstructive sleep apnea were screened for eligibility. Clinical suspicion of obstructive sleep apnea was based on symptoms and/or signs suggestive of OSA, including habitual snoring, witnessed apneas, nocturnal choking, excessive daytime sleepiness, morning headache, and other clinically relevant indications prompting overnight polysomnography.

According to the standards of the American Academy of Sleep Medicine (AASM), OSA was diagnosed by nocturnal polysomnography (PSG) as an apnea–hypopnea index (AHI) of ≥5 events per hour in the presence of compatible symptoms. OSA severity was classified as mild (AHI 5.0–14.9 events/h), moderate (15.0–29.9 events/h), or severe (≥30.0 events/h) [9,10].

Patients with polysomnography-confirmed OSA were considered for inclusion. Hypertension was defined as systolic blood pressure ≥ 140 mmHg and/or diastolic blood pressure ≥ 90 mmHg on repeated office measurements, or current use of antihypertensive medication. Among these patients, those eligible for PA evaluation according to the institutional workflow underwent further PA assessment. OSA patients who did not enter the PA evaluation pathway were not included in the PA-related analytic cohort. All consecutive eligible patients with definitive PA classification during the study period were included and categorized into the OSA+PA or OSA-only group. No a priori sample-size calculation was performed.

PA was diagnosed according to the Clinical Practice Guidelines of the Endocrine Society. PA evaluation followed a stepwise institutional workflow. Baseline biochemical screening included plasma aldosterone concentration, direct renin concentration (DRC), and the aldosterone-to-renin ratio (ARR). ARR was calculated as the ratio of plasma aldosterone concentration to DRC. Confirmatory testing, including saline infusion testing and/or captopril challenge testing, was performed when clinically indicated and selected according to screening results, clinical suitability, contraindications, and physician judgment. Therefore, not all patients underwent both confirmatory tests. Patients without sufficient PA evaluation to determine definitive PA status were excluded from the final analytic cohort. Final PA diagnosis in this study was based on biochemical findings, confirmatory testing, and adrenal imaging, according to the institutional clinical workflow [8].

Exclusion criteria included: (1) other causes of secondary hypertension, such as renal artery stenosis, Cushing’s syndrome, or pheochromocytoma; (2) severe cardiac, hepatic, or renal insufficiency; (3) recent use of drugs that interfere with the renin–angiotensin–aldosterone system, including mineralocorticoid receptor antagonists, ACE inhibitors, angiotensin receptor blockers, or diuretics, without a sufficient washout period; (4) previous treatment for obstructive sleep apnea (such as continuous positive airway pressure) or inability to undergo standard nocturnal polysomnography; and (5) pregnancy, active malignancy, systemic inflammatory disease, or any other condition deemed by the investigators to affect the validity of the study.

### 2.2. Clinical and Biochemical Indicators

Standardized methods were used to obtain anthropometric measurements, including body mass index (BMI), neck circumference, waist circumference, and hip circumference. All circumferences were measured with a non-stretchable tape while the participant was standing, and neck circumference was measured at the mid-neck level. After fasting for at least 8 h, a baseline fasting venous blood sample was collected in the morning at the initial biochemical assessment, before PA confirmatory testing when performed and before initiation of PA-directed treatment. Serum potassium, creatinine, uric acid, fasting blood glucose, glycated hemoglobin (HbA1c), and blood lipids were determined in the hospital laboratory by automated enzymatic methods. Plasma aldosterone was measured using chemiluminescence immunoassay (LIAISON XL, DiaSorin, Italy). Direct renin concentration (DRC) was measured as part of the PA screening workup, and ARR was calculated as the ratio of plasma aldosterone concentration to DRC. Quality control was performed daily as recommended by the manufacturer. If the test was conducted within 5 days, the sample was stored at 2–8 °C; otherwise, it was frozen at −20 °C. A maximum of three freeze–thaw cycles was allowed.

### 2.3. Polysomnography

All participants underwent overnight laboratory PSG recordings using a standardized system (SOMNOscreen, SOMNOmedics GmbH, Randersacker, Germany). The recorded signals included electroencephalogram, electrocardiogram, electromyogram, airflow (nasal pressure sensor and thermistor), chest and abdominal movements, arterial oxygen saturation (SpO_2_), and snoring signal. Experienced technicians manually scored PSG records based on the 2012 AASM scoring manual, and the apnea–hypopnea index (AHI), oxygen desaturation index (ODI), minimum oxygen saturation, and snoring index were extracted for analysis [11].

### 2.4. Statistical Analysis

Continuous variables were assessed for normality using the Shapiro–Wilk test. Normally distributed continuous variables are presented as mean ± standard deviation (SD), whereas non-normally distributed continuous variables are reported as median (interquartile range, IQR). Categorical variables are expressed as counts and percentages. Group comparisons were performed using Student’s *t*-test or Mann–Whitney U test for continuous variables and the chi-square test or Fisher’s exact test for categorical variables, as appropriate.

Pearson correlation and PA status–adjusted partial correlation analyses were used to assess associations between biochemical variables and polysomnographic parameters. Multivariable logistic regression analysis was performed to identify factors independently associated with PA among patients with OSA, with model calibration assessed by the Hosmer–Lemeshow test and variance inflation factors (VIFs) used to assess multicollinearity. Statistical significance was set at two-tailed *p* < 0.05. Analyses were conducted using SPSS version 25.0 (IBM, Armonk, NY, USA) and Stata 17.0 (StataCorp, College Station, TX, USA).

## 3. Results

### 3.1. Comparison of the Main Clinical Characteristics of the Study Population

**Figure 1:**
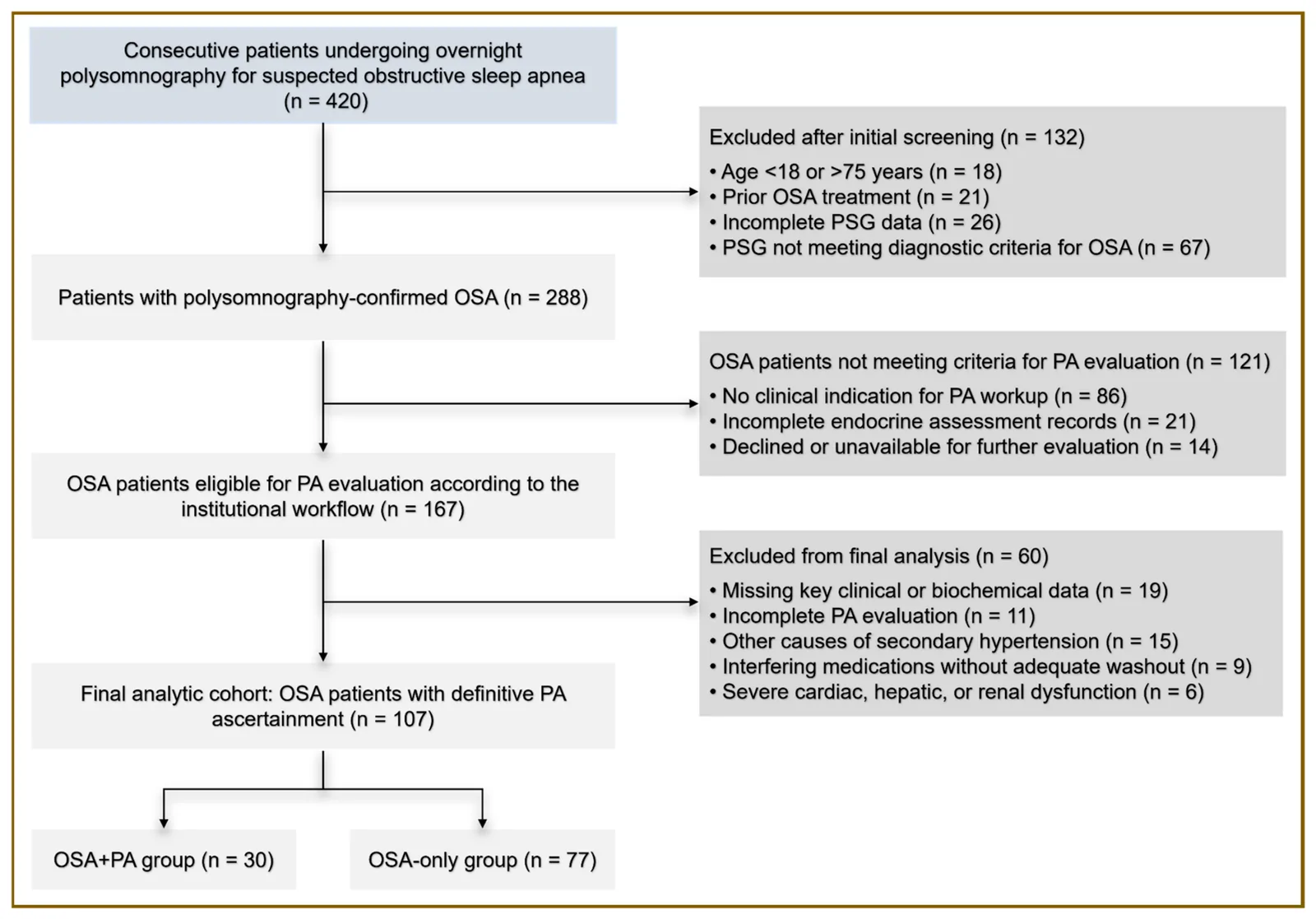
Flowchart of participant selection, PA evaluation, and final grouping in patients with obstructive sleep apnea.

The participant selection process is shown in Figure 1. A total of 107 patients with definitive PA classification were included in the final analysis, including 30 patients in the OSA+PA group and 77 patients in the OSA-only group. Baseline clinical and polysomnographic characteristics are summarized in Table 1. There were no significant differences in the main clinical characteristics between the two groups, including gender, age, BMI, waist and hip circumference (*p* > 0.05). However, in terms of neck circumference, the OSA+PA group showed a greater value than the OSA-only group (42.0 ± 4.6 cm vs. 39.8 ± 3.7 cm, *p* = 0.010). In addition, among polysomnographic variables, only OSA severity and snoring index (369.9 ± 320.3 events/h vs. 252.0 ± 264.9 events/h, *p* = 0.040) differed significantly between the two groups. The remaining parameters, including AHI, ODI, apnea index, hypopnea index, nocturnal hypoxemia, maximum apnea duration, and oxygen saturation measures, showed no statistically significant differences (*p* > 0.05).

### 3.2. Comparison of Biochemical Characteristics of the Study Population

Several biochemical parameters differed significantly between the two groups (Table 2). Serum aldosterone was significantly higher in the OSA+PA group than in the OSA-only group (25.8 ± 18.8 vs. 10.7 ± 7.3 ng/dL, *p* = 0.001). Serum potassium was significantly lower in the OSA+PA group than in the OSA-only group (3.5 ± 0.4 vs. 4.0 ± 0.4 mmol/L, *p* = 0.001). HbA1c was also lower in the OSA+PA group (6.4 ± 1.6% vs. 7.5 ± 2.1%, *p* = 0.005). No significant between-group differences were observed in the remaining biochemical parameters, including uric acid, creatinine, liver enzymes, lipid profile, vitamin D, PTH, and thyroid function (all *p* > 0.05).

### 3.3. Logistic Regression Analysis

To examine factors associated with coexisting PA among patients with OSA, we conducted a multivariable logistic regression analysis. We included neck circumference, snoring index, aldosterone, glycated hemoglobin and serum potassium in the multivariate logistic regression. The overall fit of the model was good (Pseudo R^2^ = 0.454; LLR test *p* < 0.001). Multicollinearity among the variables included in the multivariable logistic regression model was assessed using variance inflation factors. The VIF values ranged from 1.062 to 1.262, indicating no substantial multicollinearity among the included covariates. The results showed that serum potassium was significantly negatively associated with coexisting PA among patients with OSA (OR = 0.017, *p* = 0.001), and aldosterone was significantly positively associated with coexisting PA among patients with OSA (OR = 1.138, *p* = 0.001). However, the snoring index was marginally associated with coexisting PA (*p* = 0.087), whereas neither neck circumference nor HbA1c was significantly associated with coexisting PA (both *p* > 0.05) (Table 3). These findings indicate that higher serum aldosterone and lower serum potassium remained associated with coexisting PA in the multivariable model.

Furthermore, considering the uneven distribution of diabetes between the two groups (59.7% in the OSA group and 23.3% in the OSA+PA group, *p* = 0.002), we further included HbA1c and diabetes status as covariates to construct a logistic regression model for adjustment. The results (Table A1) showed that aldosterone (OR = 1.127, *p* = 0.002) and serum potassium (OR = 0.032, *p* < 0.001) remained associated with coexisting PA, while HbA1c or diabetic status did not show significance.

### 3.4. Correlation Between Aldosterone and Sleep Parameters

**Figure 2:**
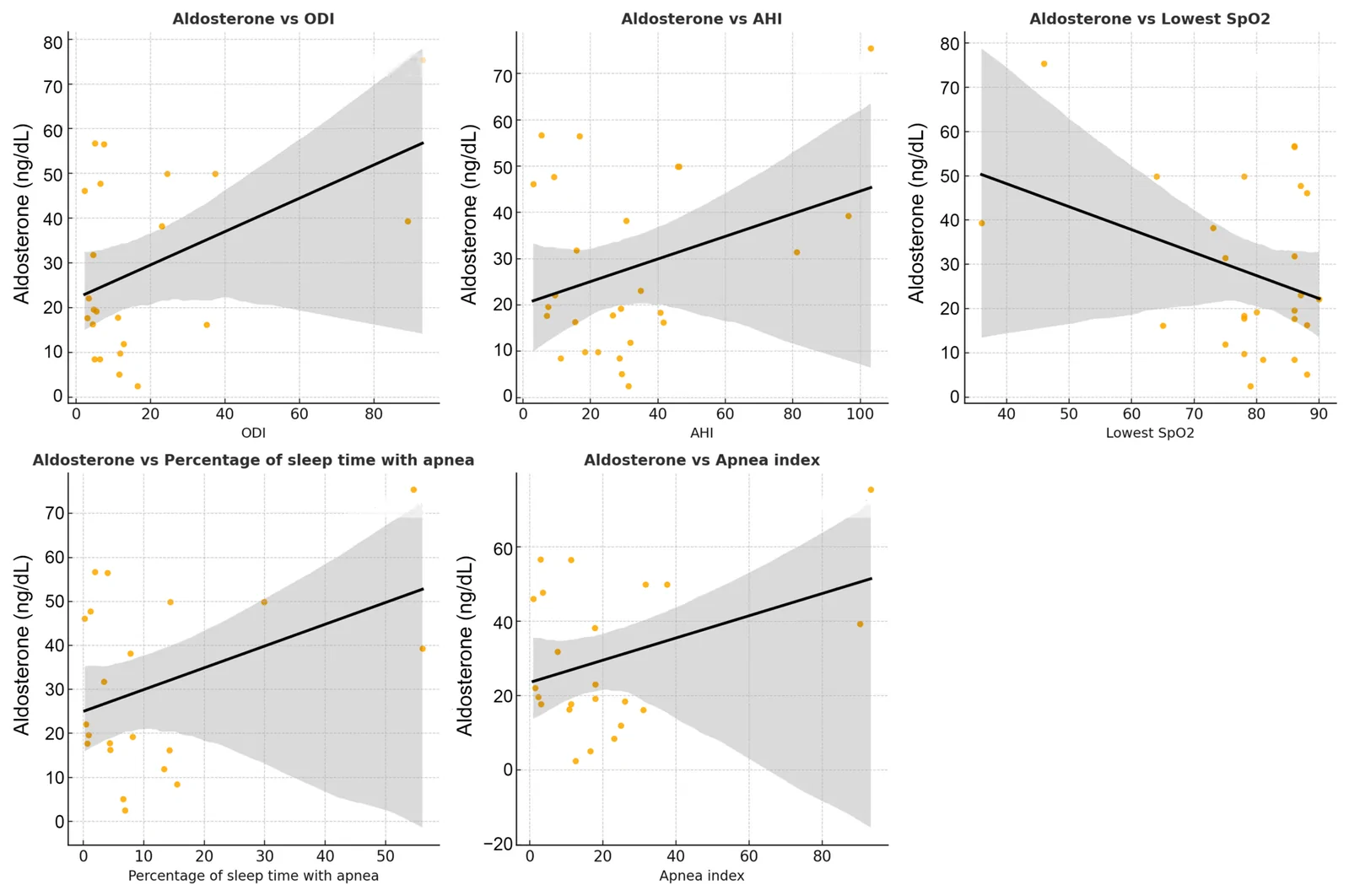
Correlation between aldosterone levels and polysomnographic parameters. Scatter plots demonstrate the correlations between serum aldosterone and key polysomnographic variables, including apnea–hypopnea index (AHI), oxygen desaturation index (ODI), apnea index, percentage of sleep time with apnea, and minimum oxygen saturation (SpO_2_). Solid lines represent fitted regression lines, and the shaded areas indicate 95% confidence intervals.

In the pooled cohort, serum aldosterone was positively correlated with AHI, ODI, percentage of sleep time with apnea, and apnea index, and negatively correlated with lowest SpO_2_ (Table 4 and Figure 2). After adjustment for PA status, the correlations with ODI (partial r = 0.266, *p* = 0.006), AHI (partial r = 0.223, *p* = 0.022), apnea index (partial r = 0.211, *p* = 0.030), lowest SpO_2_ (partial r = −0.225, *p* = 0.020), and percentage of sleep time with apnea (partial r = 0.182, *p* = 0.042) remained significant.

## 4. Discussion

The association between PA and OSA has become an important focus of clinical and basic research in recent years. Substantial epidemiological and clinical evidence indicates that the prevalence of OSA is significantly higher in patients with PA, and that OSA can further exacerbate elevated blood pressure and target-organ damage caused by PA [2]. However, the clinical features associated with coexisting PA in patients with OSA remain incompletely characterized. We therefore evaluated clinical, polysomnographic, and biochemical parameters associated with coexisting PA among patients with OSA.

In this cohort, higher serum aldosterone and lower serum potassium in patients with OSA and coexisting PA were consistent with the expected biochemical phenotype of PA. Among the other biochemical variables, HbA1c was also lower in the OSA+PA group; however, this difference should be interpreted cautiously because diabetes was less prevalent in this group and HbA1c was not independently associated with coexisting PA in the extended model. It may therefore reflect the imbalance in diabetes prevalence rather than a PA-specific metabolic feature. Correlation analysis further showed that serum aldosterone was associated with several polysomnographic parameters. However, because aldosterone contributed to PA classification and hypokalemia is a classic manifestation of aldosterone excess, the biochemical findings primarily confirm the established PA phenotype rather than identify novel independent predictors.

Obesity may represent a shared upstream factor linking OSA and aldosterone excess. Previous studies showed that BMI predicted aldosterone production in normotensive adults receiving a high-sodium diet [12] and plasma aldosterone concentrations in patients with primary hypertension, particularly those with overweight or obesity [13]. Experimental evidence further demonstrated that adipocyte-derived leptin can directly stimulate adrenal aldosterone synthesis and secretion [14]. Because obesity is also a major risk factor for OSA, an adiposity–leptin–aldosterone pathway is biologically plausible. However, BMI and waist circumference did not differ significantly between the OSA+PA and OSA-only groups in our cohort. Thus, general adiposity did not appear to explain the observed between-group differences, although visceral adiposity and circulating leptin were not measured.

Hypokalemia, as a classic biochemical feature of PA, also showed important clinical relevance in this study. The decline in serum potassium levels is not only a direct result of excessive aldosterone secretion leading to sodium retention and potassium loss in the distal convoluted tubules and collecting ducts, but also may contribute to the occurrence and progression of OSA through multiple pathways [15]. On the one hand, hypokalemia can affect the resting potential and action potential conduction of striated muscle cell membranes, leading to decreased contractility of the respiratory and upper airway muscles [16], which may make patients more prone to airway collapse and ventilation disorders during sleep. On the other hand, chronic hypokalemia often coexists with metabolic alkalosis, which can exacerbate abnormal central nervous system sensitivity to changes in carbon dioxide and oxygen partial pressures, thereby further promoting respiratory instability [17,18]. Therefore, our findings further support the clinical relevance of hypokalemia as a clue to coexisting PA in patients with OSA, especially in those whose blood pressure is difficult to control or who experience substantial nocturnal hypoxia, which warrants greater attention.

Similarly, as a core effector molecule of the renin–angiotensin–aldosterone system (RAAS), aldosterone is regarded as a pivotal molecule that plays a key role in the mutual promotion between OSA and PA, jointly leading to cardiovascular damage. On the one hand, the continuous excessive secretion of aldosterone in patients with PA not only causes sodium retention, fluid accumulation, and blood volume expansion, but also promotes edema of the upper airway mucosa and surrounding tissues, thereby increasing the susceptibility to airway collapse and exacerbating the severity of OSA [2]. In addition, aldosterone can induce local inflammatory responses and oxidative stress, damage vascular endothelial function, and enhance sympathetic nervous system activity. This may amplify the pathological effects caused by the characteristic intermittent hypoxia of OSA, leading to a more significant increase in blood pressure variability [19]. On the other hand, OSA patients themselves can drive abnormal activation of the RAAS through repeated hypoxia and reoxygenation processes as well as excessive activation of the nervous system at night, which in turn stimulates the secretion of aldosterone, thus forming a self-reinforcing vicious cycle. In this cycle, aldosterone is not only a key factor driving the continuous increase in blood pressure, but also directly participates in the processes of multiple target organ damage, such as myocardial hypertrophy, vascular remodeling, and kidney injury [20,21]. Moreover, relevant studies have also reported that the plasma aldosterone concentration in PA patients with coexisting OSA is usually significantly higher, and the severity of OSA (such as AHI) is positively correlated with aldosterone levels [22]. It is worth noting that the use of aldosterone antagonists (such as spironolactone and eplerenone) not only improves blood pressure control but also reduces the AHI in patients with OSA to a certain extent [23,24], further suggesting that aldosterone plays a core role in the pathological interaction between the two.

Interestingly, this study found that aldosterone levels were positively correlated with both AHI and ODI, and the correlation with ODI was stronger. Previous animal and clinical studies have shown that intermittent hypoxia can promote aldosterone production by activating the sympathetic nervous system and increasing renin secretion [19,25]. In contrast, excessive aldosterone can cause fluid retention and rostral fluid redistribution, leading to laryngeal edema and thereby aggravating OSA severity, thereby forming a vicious cycle [26]. This bidirectional mechanism provides a plausible explanation for why OSA patients with PA often exhibit a more severe nocturnal hypoxic load, and it is highly consistent with our finding that aldosterone levels are independently associated with ODI.

Furthermore, we noted that relying solely on polysomnography parameters (such as AHI and ODI) to distinguish whether OSA patients have concurrent PA has limited discriminative value. In contrast, the snoring index has some indicative value. The potential cause may be that snoring directly reflects the mechanical changes in upper airway obstruction, and fluid retention caused by excessive aldosterone associated with PA may more easily cause local structural abnormalities, such as tissue edema in the pharynx [27,28], thereby intensifying snoring. However, its predictive performance is still significantly lower than that of biochemical indicators such as aldosterone and blood potassium, suggesting that the PSG parameters are more suitable as a supplement rather than a core tool in screening.

This study has several limitations. First, the sample size was limited, with only 30 patients in the OSA+PA group, which reduced the precision and stability of the regression estimates. Second, as a cross-sectional study, causality cannot be inferred. In addition, because aldosterone contributed to PA classification and hypokalemia may have influenced referral for PA evaluation, associations involving these variables are susceptible to incorporation bias and should not be interpreted as independent diagnostic effects. Third, several potential confounders were incompletely captured in this retrospective dataset, including long-term antihypertensive medication exposure, drug class, treatment duration and burden, sodium intake, the duration and severity of hypertension, and renal function markers beyond serum creatinine. Although patients taking renin–angiotensin–aldosterone system-interfering medications without an adequate washout period were excluded, residual effects of prior long-term treatment on renin, aldosterone, and serum potassium cannot be excluded. Finally, because the source population comprised patients with polysomnography-confirmed OSA, no PA-only group was included, limiting assessment of the independent contributions of PA and OSA. External validation in larger, multicenter cohorts is required.

## 5. Conclusions

In this clinically selected cohort, coexisting PA in patients with OSA was characterized predominantly by its biochemical phenotype of higher aldosterone and lower serum potassium, whereas most anthropometric and polysomnographic measures showed limited between-group differences. These findings indicate that sleep-study characteristics alone are unlikely to reliably distinguish coexisting PA and reinforce the importance of guideline-based biochemical evaluation when PA assessment is clinically indicated. Larger prospective multicenter studies incorporating direct measures of adiposity and related endocrine mediators are needed to clarify the biological links among OSA, obesity, and aldosterone excess.

## Figures and Tables

**Table 1:** Comparison of clinical characteristics between the OSA+PA and OSA groups.

Variable	OSA+PA Group (n = 30)	OSA Group (n = 77)	*p* Value
Sex, n (%)			0.316
Male	18 (60.0)	54 (70.1)	
Female	12 (40.0)	23 (29.9)	
Age, years	46.3 ± 12.4	47.5 ± 15.5	0.690
BMI, kg/m^2^	26.7 ± 4.5	27.8 ± 4.4	0.254
Waist circumference, cm	97.7 ± 11.9	98.3 ± 9.3	0.997
Hip circumference, cm	101.6 ± 11.1	102.7 ± 8.5	0.870
Neck circumference, cm	42.0 ± 4.6	39.8 ± 3.7	0.010
Hypertension, n (%)			0.020
Yes	30 (100.0)	56 (72.7)	
No	0 (0.0)	21 (27.3)	
OSA severity, n (%)			0.015
Mild	11 (36.7)	12 (15.6)	
Moderate	7 (23.3)	39 (50.6)	
Severe	12 (40.0)	26 (33.8)	
Nocturnal hypoxemia, n (%)			0.850
None	2 (6.7)	3 (3.9)	
Mild	13 (43.3)	30 (39.0)	
Moderate	7 (23.3)	20 (26.0)	
Severe	8 (26.7)	24 (31.2)	
AHI, events/h	32.0 ± 29.4	28.5 ± 19.1	0.622
Maximum apnea duration, s	38.0 ± 17.2	39.1 ± 18.3	0.744
Baseline SpO_2_, %	95.9 ± 2.2	95.6 ± 2.3	0.459
Lowest SpO_2_, %	77.9 ± 14.8	79.3 ± 11.5	0.641
Highest SpO_2_, %	99.4 ± 1.2	99.7 ± 0.6	0.230
Mean SpO_2_, %	94.5 ± 1.8	94.3 ± 2.0	0.769
Percentage of sleep time with apnea, %	15.4 ± 22.0	10.1 ± 13.8	0.313
Percentage of sleep time with hypopnea, %	5.7 ± 3.5	7.0 ± 4.0	0.085
Apnea index, events/h	20.8 ± 25.9	16.9 ± 17.8	0.535
Hypopnea index, events/h	9.1 ± 5.7	11.3 ± 7.0	0.115
Snoring index, events/h	369.9 ± 320.3	252.0 ± 264.9	0.040
ODI, events/h	23.7 ± 29.9	17.0 ± 16.7	0.591

Note: Data are presented as mean ± standard deviation (SD), median (interquartile range, IQR), or n (%), as appropriate. *p* values were calculated using Student’s *t*-test, Mann–Whitney U test, chi-square test, or Fisher’s exact test, as appropriate. Normality was assessed using the Shapiro–Wilk test. Abbreviations: OSA, obstructive sleep apnea; PA, primary aldosteronism; BMI, body mass index; AHI, apnea–hypopnea index; ODI, oxygen desaturation index; SpO_2_, oxygen saturation.

**Table 2:** Comparison of serum biochemical parameters between OSA+PA and OSA groups.

Variable	PA+OSA Group (n_1_ = 30)	OSA Group (n_2_ = 77)	*p* Value
Aldosterone (ng/dL)	25.8 ± 18.8	10.7 ± 7.3	0.001
HbA1c (%)	6.4 ± 1.6	7.5 ± 2.1	0.005
Serum potassium (mmol/L)	3.5 ± 0.4	4.0 ± 0.4	0.001
Uric acid (μmol/L)	389.8 ± 119.1	391.1 ± 93.2	0.953
Creatinine (μmol/L)	84.5 ± 42.4	95.8 ± 77.4	0.369
ALT (U/L)	28.2 ± 21.2	27.8 ± 10.8	0.117
AST (U/L)	29.6 ± 6.1	27.5 ± 7.2	0.134
Total cholesterol (mmol/L)	4.6 ± 1.2	5.0 ± 1.4	0.293
Triglycerides (mmol/L)	2.5 ± 2.0	2.7 ± 2.5	0.535
HDL-C (mmol/L)	1.1 ± 0.3	1.1 ± 0.2	0.685
LDL-C (mmol/L)	3.0 ± 0.8	3.2 ± 0.9	0.306
Vitamin D (ng/mL)	20.6 ± 4.9	21.8 ± 8.2	0.555
PTH (pg/mL)	61.0 ± 36.4	56.3 ± 23.9	0.861
FT3 (pmol/L)	4.8 ± 0.6	4.4 ± 0.9	0.060
FT4 (pmol/L)	16.1 ± 2.2	15.2 ± 2.9	0.243
TSH (mIU/L)	2.5 ± 1.9	3.0 ± 4.4	0.602

Note: Data are presented as mean ± SD, median (IQR), or n (%). *p* values were calculated using Student’s *t*-test, Mann–Whitney U test, chi-square test, or Fisher’s exact test, as appropriate. Normality was tested using the Shapiro–Wilk test. Abbreviations: HbA1c, glycated hemoglobin; ALT, alanine aminotransferase; AST, aspartate aminotransferase; HDL-C, high-density lipoprotein cholesterol; LDL-C, low-density lipoprotein cholesterol; PTH, parathyroid hormone; FT3, free triiodothyronine; FT4, free thyroxine; TSH, thyroid-stimulating hormone.

**Table 3:** Multivariable logistic regression analysis of factors associated with coexisting PA among patients with OSA.

Variable	β	SE	OR	95% CI Lower	95% CI Upper	*p* Value	VIF
Neck circumference	−0.016	0.050	0.984	0.892	1.085	0.744	1.062
Snoring index	0.002	0.001	1.002	1.000	1.003	0.087	1.063
Aldosterone	0.130	0.040	1.138	1.051	1.231	0.001	1.207
HbA1c	−0.126	0.181	0.882	0.618	1.258	0.488	1.167
Serum potassium	−4.097	0.977	0.017	0.002	0.113	0.001	1.262

Note: Variables entered into the model included neck circumference, snoring index, aldosterone, HbA1c, and serum potassium. The table presents regression coefficients (β), standard errors (SEs), odds ratios (ORs), 95% confidence intervals (CIs), *p* values, and VIF values. VIF values were calculated to assess multicollinearity among the variables included in the multivariable logistic regression model. Aldosterone and serum potassium were significantly associated with coexisting PA. Abbreviations: HbA1c, glycated hemoglobin; OR, odds ratio; CI, confidence interval; SE, standard error; VIF, variance inflation factor.

**Table 4:** Correlation analysis between aldosterone and polysomnographic parameters.

Variable	Unadjusted r	Unadjusted *p* Value	PA-Adjusted Partial r	Adjusted *p* Value
Hypopnea index	−0.180	0.063	−0.126	0.199
Snoring index	0.021	0.830	−0.087	0.377
ODI	0.299	0.002	0.266	0.006
AHI	0.227	0.018	0.223	0.022
Maximum apnea duration	0.088	0.368	0.119	0.225
Baseline SpO_2_	0.028	0.772	−0.003	0.974
Lowest SpO_2_	−0.218	0.024	−0.225	0.020
Highest SpO_2_	−0.011	0.912	0.092	0.349
Mean SpO_2_	−0.056	0.566	−0.090	0.359
Percentage of sleep time with apnea	0.191	0.028	0.182	0.042
Percentage of sleep time with hypopnea	−0.202	0.037	−0.150	0.126
Apnea index	0.225	0.020	0.211	0.030

Note: Pearson correlation coefficients and partial correlation coefficients adjusted for PA status are presented. Abbreviations: AHI, apnea–hypopnea index; ODI, oxygen desaturation index; SpO_2_, oxygen saturation.

## Data Availability

The data presented in this study are available upon request from the corresponding author.

## Abbreviations

The following abbreviations are used in this manuscript:

AASM	American Academy of Sleep Medicine
ACE	Angiotensin-converting enzyme
AHI	Apnea–hypopnea index
ALT	Alanine aminotransferase
ARR	Aldosterone-to-renin ratio
AST	Aspartate aminotransferase
BMI	Body mass index
CI	Confidence interval
CPAP	Continuous positive airway pressure
DRC	Direct renin concentration
FT3	Free triiodothyronine
FT4	Free thyroxine
HbA1c	Glycated hemoglobin
HDL-C	High-density lipoprotein cholesterol
IQR	Interquartile range
LDL-C	Low-density lipoprotein cholesterol
LLR	Log-likelihood ratio
ODI	Oxygen desaturation index
OR	Odds ratio
OSA	Obstructive sleep apnea
PA	Primary aldosteronism
PSG	Polysomnography
PTH	Parathyroid hormone
RAAS	Renin–angiotensin–aldosterone system
SD	Standard deviation
SE	Standard error
SpO_2_	Peripheral oxygen saturation
TSH	Thyroid-stimulating hormone
VIF	Variance inflation factor

## Appendix A

**Table A1:** Logistic regression analysis: Base model vs. extended model including diabetes variables.

Variable	OR Base Model *	95% CI (Lower–Upper)	*p* Value	OR Extended Model †	95% CI (Lower–Upper)	*p* Value
Neck circumference (cm)	1.013	0.951–1.080	0.687	1.013	0.948–1.083	0.681
Snore index (events/h)	1.002	1.000–1.004	0.026	1.002	0.999–1.004	0.128
Aldosterone (ng/dL)	1.127	1.051–1.207	0.001	1.127	1.051–1.207	0.002
Serum potassium (mmol/L)	0.032	0.004–0.211	<0.001	0.032	0.004–0.233	<0.001
HbA1c (%)	–	–	–	0.964	0.665–1.398	0.837
Diabetes (yes vs. no)	–	–	–	0.812	0.210–3.142	0.763

Note: The base model included neck circumference, snoring index, aldosterone, and serum potassium. The extended model additionally incorporated glycated hemoglobin (HbA1c) and diabetes status. Odds ratios (ORs) with 95% confidence intervals (CIs) and *p* values are presented. In both models, aldosterone was positively associated and serum potassium was negatively associated with coexisting PA among patients with OSA, while HbA1c and diabetes status were not significantly associated with coexisting PA. * Base model included neck circumference, snoring index, aldosterone, and serum potassium. † Extended model additionally included HbA1c and diabetes status.
